# Nutritional characterization and food value addition properties of dehydrated spinach powder

**DOI:** 10.1002/fsn3.2110

**Published:** 2021-01-07

**Authors:** Muhammad Waseem, Saeed Akhtar, Muhammad Faisal Manzoor, Asif A. Mirani, Zulfiqar Ali, Tariq Ismail, Nazir Ahmad, Emad Karrar

**Affiliations:** ^1^ Institute of Food Science & Nutrition Bahauddin Zakariya University Multan Pakistan; ^2^ School of Food and Biological Engineering Jiangsu University Zhenjiang China; ^3^ Postharvest and Food Eng. Agricultural Engineering Division Islamabad Pakistan; ^4^ Institute of Home and Food Sciences Government College University Faisalabad Pakistan; ^5^ Department of Food Engineering Faculty of Engineering University of Gezira Wad Medani Sudan

**Keywords:** dough rheology, spinach powder, supplementation, unleavened bread, wheat flour

## Abstract

This study aimed at investigating the physicochemical and bread‐making features of dehydrated spinach. Physicochemical composition of spinach powder was compared with wheat flour and the effect of spinach powder supplementation on the nutritional composition, dough rheology, and quality attributes of *chapatti* were assessed. The results suggested spinach powder to be holding 8.2% crude fiber, 19.2% protein, 1,304 mg/100g calcium, and 40.4 mg/100g iron. Spinach powder indicated significantly increased values for hygroscopicity, swelling power, and water solubility index values, that is, 6.4%, 7.1 g/g, and 4.2%, respectively, when compared with wheat flour. Supplementation of spinach powder in wheat flour at 20% substitution level significantly reduced dough development properties including water absorption, dough stability, and peak dough development time. Color measurements of baked *chapatti* indicated a significant reduction in L*, a*, and chroma values with increasing the level of spinach powder supplementation; however, sensory profiling confirmed that supplementation of spinach powder at 7.5% had an optimum effect on the overall acceptability of the baked product. The results further suggested that replacing wheat flour with spinach powder (5%–7.5%, w/w) in baked products could be a viable dietary approach to enhance the optimum supply of micronutrients and to combat micronutrient deficiencies among various population segments.

## Graphical abstract

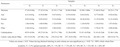

## 1 INTRODUCTION

Spinach (*Spinacia oleracea* L.), a dark green leafy vegetable, belongs to the family Chenopodiaceae (Li et al., 2019) and is extensively consumed across the globe on account of its unique nutritional composition. Spinach stands out as one of the naturally enriched vegetables that hold an array of phytonutrients and bioactive compounds like β‐carotene, lutein, zeaxanthin, ascorbic acid, flavonoids, and polyphenols with good biological properties (Manzoor et al., 2020; Salehi et al., 2019). Spinach is also referred to as a cheaper source of dietary fibers and minerals including calcium, phosphorus, potassium, magnesium, iron, zinc, copper, and manganese. Likewise, spinach has been considered as a potential source of carotenoids, folates, and iron and elicits ameliorative impacts against eye disorders like macular degeneration, nutritional anemia, and neural tube defects (Edelman & Colt, 2016; Shaheen et al., 2018). Spinach leaves also contain exceptionally higher amounts of phylloquinone that serves as a remedy to bleeding disorders (Edelman & Colt, 2016).

World Health Organization (WHO) estimated 190 million preschool children to be the victims of deficiency of one or more micronutrients including vitamin A and iron (Paul et al., 2017). Available information suggests increased production of food crops as a means to bridge up inherent nutritional inadequacies and losses. In addition to food fortification, dietary diversification serves as a sustainable approach to subside micronutrient malnutrition and related health challenges (Lindsay et al., 2006). Staple food crops and the products made thereof are recommended as potential means to mitigate micronutrient deficiencies in developing world (Khan et al., 2015). Together with an array of bioactive compounds, spinach is regarded as a source of essential nutrients that may help in preventing a variety of nutritional disorders. Cereals based bakery products are the most common, viable, and acceptable consumable goods in various cultures. Sufficient literature demonstrates the application of vegetables as natural additives to improve nutritional and functional properties of baked goods, for example, carrot pomace‐based bread and cakes (Kumar & Kumar, 2012), flaxseed powder‐based muffins (Sudha et al., 2010), fenugreek flour‐based biscuits (Hooda & Jood, 2005), and lotus seed flour‐based cookies (Shahzad et al., 2020).

*Chapattis* (unleavened flatbread) are considered as staple foods of populations in the Indian subcontinent. Different variants of leavened and unleavened flatbread include *chapatti*, *tandoori roti*, *kulcha*, *paratha*, *puri,* and *doli roti* regionally customized in various parts of Pakistan and other South Asian countries like India, Bangladesh and Afghanistan, and Iran (Gocmen et al., 2009). Around 1.8 billion people have been reported to consume flatbread as their primary source of energy in Middle Eastern regions, India, Southern Europe, Scandinavian countries, Central America, China, and South Africa (Malik et al., 2016).

A significant upsurge in nutritional inadequacies and associated health challenges necessitates the development of recipes and food formulations exploiting nonconventional functional ingredients. The present study aimed to investigate and evaluate the potential for supplementation of spinach powder in wheat flour and its effect on textural and sensory properties to *chapattis* made thereof. The study further highlights various functional properties and nutritional significance of the vegetable powder supplemented chapattis suggesting wheat flour value addition a viable approach to mitigating food and nutritional insecurity among regional folk.

## 2 MATERIALS AND METHODS

### 2.1 Raw materials and chemicals

Fresh whole spinach leaves (20 kg) and whole wheat flour (20 kg) were procured from the vegetable and grain markets of Multan, Pakistan, respectively. All the chemicals and reagents used in the physicochemical analysis were of analytical grade and procured from Merck and Sigma Chemical Co., Ltd. unless otherwise mentioned in this study.

### 2.2 Preparation of spinach powder (SP)

Fresh leaves of spinach were subjected to preliminary operations such as sorting, grading, washing, and dirt removal. Leaves were dipped in 40 ppm sodium hypochlorite (NaOCl_3_) solution for 30 min. Residues of sodium hypochlorite were removed by subsequent washing of shredded spinach leaves with potable water. Drying of processed spinach leaves was carried out to 15%–17% moisture contents in a cabinet dryer (PAKFVS‐40T) at 45 ± 2°C. Dehydrated leaves were ground to 72 mm mesh size in a heavy‐duty grinder. Spinach powder thus obtained was packed in airtight polyethylene bags and stored at 4–6 ± 2°C for further experimentation (Galla et al., 2017).

### 2.3 Functional properties of wheat flour (WF) and spinach powder (SP)

#### 2.3.1 Bulk density (BD)

Spinach powder and wheat flour samples were evaluated for bulk density by the method followed by Jan et al. (2015). Briefly, 100g of each sample was poured into the tarred graduated cylinders (500 ml). The cylinder was tapped twice to fill any of the remaining space for homogeneity. BD was estimated using the following formula:

$$\text{Bulk Density}\;\left(g/\text{ml}\right)=\text{Sample weight}\;\left(g\right)/\text{Volume}\text{occupied by sample}\;\left(\text{ml}\right).$$

#### 2.3.2 Rehydration Ratio (RR)

The rehydration ratio was determined by the method followed by Shaari et al. (2018). Five grams of each sample was soaked into 50 ml distilled water for 60 min at room temperature. Soaked samples were filtered using Whatman filter paper No. 41, and permeate was weighed. Samples’ ability to absorb water or rehydrate upon soaking was measured using the following formula:

$$\text{Rehydration ratio}\;=\;\text{Weight of drained material}\;\left(g\right)\;÷\;\text{Weight of dried residues}\;\left(g\right).$$

#### 2.3.3 Water absorption capacity (WAC)

The water absorption capacity of the samples was estimated following the method followed by Shafi et al. (2016). One gram of each sample was mixed in 10 ml of distilled water, and the mixture was subjected to incubation of 30 min. Thereafter, each sample was centrifuged (Hermle Z236K) at 2000 rpm for 10 min. The supernatant was decanted, and residues were inverted onto the filter paper to allow water to be released for 5 min. WAC was estimated by weighing the residues and represented as the percentage of water absorbed per gram of the sample.

#### 2.3.4 Water solubility index (WSI)

The water solubility index was determined by following the method of Asaduzzaman et al. (2013). One gram of each sample was thoroughly mixed with 10 ml distilled water. The mixture was centrifuged (Hermle Z236K) for 30 min at 4,000 rpm. The supernatant thus obtained was oven‐dried (Memmert) at 70 ± 5°C till the constant weight. Dried samples were cooled in the desiccator and weighed. WSI was calculated by the following formula:

$$\text{WSI}\;\left(\%\right)\;=\;W1‐\;W2÷\;W\;*100.$$

*W* = Dried sample weight; *W*1 = Weight of petri dish and dried liquid; *W*2 = Weight of empty petri dish.

#### 2.3.5 Swelling power (S_P_)

Swelling power was determined by the method outlined by Shafi et al. (2016). Samples weighing 0.5 g each were dispersed in 50 ml distilled water. The mixture was heated for 30 min at 90°C. Centrifugation of the homogenate was performed at 3,000 rpm for 15 min. Thereafter, the supernatant was decanted and oven‐dried (Memmert) at 100 ± 5°C till constant weight. Results for S_P_ were represented in g/g of the dried sample.

#### 2.3.6 Hygroscopicity assay

Hygroscopicity values of the samples were determined by the method of Jaya and Das (2004). Each sample (10 g) was added to a petri dish and shifted to a desiccator containing sodium sulfate (Na_2_SO_4_) maintaining saturation level with approx. 81% relative humidity for one week. Results were calculated by determining differences in weight and expressed as g of moisture absorbed per 100g of the dry matter (g/100g) using the formula as mentioned below:

$$\text{Hygroscopicity}\;=\Delta m/\left(m+m_{1}\right)/1+\left(\Delta m/m\right).$$

Where *∆m* shows the increase of powder's weight at equilibrium (*g*), *m* is the primary mass of the powder (*g*), where *m*_1_ is the water‐free moisture of powder before exposure to external air humidity (g/100 g).

### 2.4 Determination of nutritional composition

Moisture (Method No. 925.10), crude protein (Method No. 920.87), crude fat (Method No. 920.85), crude ash (Method No. 923.03), crude fiber (Method No. 32–10), and carbohydrate contents of all samples were determined using the following formula: carbohydrates (%) = 100 – (moisture %+ ash% + protein%+ fat %+ fiber%) (Latimer, 2019). Mineral contents were estimated using atomic absorption spectrophotometer (Thermo Scientific iCE 3,000 series) and flame photometer (410, Sherwood Scientific Ltd‐UK) (Latimer, 2019). Caloric contents of the samples were calculated by using the following formula:

$$\text{Energy}\;\left(\text{Kcal}/g\right)\;=\;\left(\text{Proteins}\;\left(g\right)\;\times 4.00\right)\;+\;\left(\text{Fats}\;\left(g\right)\;\times 9.00\right)\;+\;\left(\text{Carbohydrates}\;\left(g\right)\;\times 4.00\right)\;\left(\text{Galla}et\;al.,2017\right).$$

### 2.5 Preparation of *chapattis* premixes

Whole wheat flour *chapattis* premixes were prepared by supplementing with spinach powder at 0%–20% and were stored in airtight polyethylene bags until final preparation.

### 2.6 Rheological measurements of *chapattis* premixes

Farinographic study was conducted and samples, that is, wheat flour and spinach powder supplemented premixes, were analyzed for water absorption, dough stability time, and dough development time by using the Brabender^®^ Farinograph (Brabender GmbH and Co. KG) (Mehfooz et al., 2018). AACC (2000) method 54–40 was followed to determine mixographic measurements, that is, peak height and the peak time of wheat flour—spinach powder preblends using Brabender^®^ Mixograph (National Mfg. Co).

### 2.7 Preparation of *chapattis*

Wheat flour and spinach powder premixes were mixed in an optimum amount of water for developing composite *chapattis*. The dough was divided manually into dough balls each weighing 50 g. Dough balls were allowed to rest for 20 min and sheeted into 150 mm diameter *chapattis* with a roller pin. *Chapattis* were baked at 225 ± 5°C for 1.5–2.5 min (Kundu et al., 2019).

### 2.8 Color, texture, and puffed height assessment

The color profile of freshly produced *chapattis* was determined by the method followed by Pekmez and Yılmaz (2018). Physical color parameters L* (brightness), a* (redness), and b* (yellowness), hue angle (θ°), and chroma of *chapattis* were quantified using Hunter Lab mini‐Scan XE Plus colorimeter (Model 45/0‐L, HAL). Baked *chapattis* were evaluated for textural parameters including hardness, chewiness, and springiness by using a texture analyzer (TA.XT plus, Stable Micro Systems) (Panghal et al., 2017). Freshly baked *chapattis* were instantaneously evaluated for puffed height (cm) using a stainless steel scale following the method documented by Rao et al. (1986).

### 2.9 Sensory evaluation of supplemented *chapattis*

Sensory evaluation of spinach powder supplemented *chapattis* was conducted by a panel of experts on the 9‐Point Hedonic Scale using sensory acceptability scale from 1—dislike extremely, to 9—like extremely. *Chapattis* prepared from flour without spinach powder (control) and spinach powder were evaluated for physical appearance, color, taste, texture, folding ability, and overall acceptability (Pathania et al., 2017).

### 2.10 Statistical analysis

Each experiment was replicated twice, and means computed were presented as means ± S.D. Data collected from analyses of spinach powder, whole wheat flour, and spinach powder supplemented *chapattis* were statistically analyzed using the analysis of variance (ANOVA) technique on Statistics 8.1 (Tallahassee, FL). The level of significance among the means was analyzed by the least significance difference (LSD) at a 5% confidence interval (Steel et al., 1997).

## 3 RESULTS AND DISCUSSION

### 3.1 Functional properties of the dehydrated spinach and wheat flour

A comparison was made between the functional properties of wheat flour and spinach powder to elucidate spinach powder ability to be considered as a natural candidate for wheat flour enrichment. The results suggest significantly higher values for hygroscopicity (6.4%), swelling power (7.1 g/g), and water solubility index (4.2%) concerning spinach powder in comparison to the wheat flour (Table 1). Spinach powder holds higher swelling power and water solubility due to the presence of carbohydrates and higher amounts of dietary fibers. Earlier, a study by David et al. (2015) reported swelling power as an important functional property of starches and proteins from flours of various origins. Increased swelling power and water solubility index (WSI) have also been attributed to higher starch contents (Shafi et al., 2016). Our results were similar for swelling power (7.95 g/g) for chestnut flour (Shafi et al., 2016). Spinach powder was found to carry 120% higher protein contents than wheat flour that might have anticipated improved hygroscopicity, SP, and WSI as compared with wheat flour.

**TABLE 1:** Nutritional and functional characteristics of whole wheat flour and spinach powder

Parameter	Whole wheat flour	Spinach powder
Bulk density (g/ml)	0.62 ± 0.00a	0.60 ± 0.00a
Rehydration ratio	3.71 ± 0.07a	3.31 ± 0.01b
Water solubility index (%)	2.53 ± 0.15b	4.23 ± 0.18a
Water absorption index (g/g)	4.73 ± 01a	3.34 ± 0.00b
Swelling power (g/g)	4.11 ± 0.22b	7.05 ± 0.30a
Hygroscopicity (%)	5.74 ± 0.00b	6.43 ± 0.00a
Moisture (%)	11.11 ± 0.15a	8.26 ± 0.21b
Ash (%)	0.22 ± 0.01b	2.99 ± 0.01a
Protein (%)	8.65 ± 0.11b	19.18 ± 0.02a
Fat (%)	1.23 ± 0.02a	1.04 ± 0.03b
Fiber (%)	2.37 ± 0.18b	8.19 ± 0.02a
Carbohydrates (%)	76.43 ± 0.18a	60.34 ± 0.19b
Caloric value (Kcal 100g^−1^)	352.20 ± 0.07a	329.26 ± 0.60b

Note

Means ± S.D. Values having identical lettering in each column are nonsignificant at *p* < .05.

### 3.2 Effect of spinach powder supplementation on rheological properties of chapattis premixes

Rheological properties of composite flour were evaluated to assess the impact of spinach powder supplementation on functional properties of dough. A significant decrease (*p <* .05) in water absorption, that is, from 63% to 55% and dough stability from 13.21 min to 10.67 min was noticed at 0% – 20% spinach powder supplementation level. However, a significant increase in peak height was recorded, that is, from 48.59BU to 66.39BU at 20% supplementation of spinach powder (Table 2). Increment in dough development time, reduction in dough stability, and water absorption with increasing levels of spinach powder may be attributed to a gradual reduction in mean gluten concentration of composite flours and lesser interactions between hydroxyl groups of fibers with water (Sharma et al., 2013). Similar trends in dough development time and dough stability properties were suggested by Sharma et al. (1995) wherein researchers reported a significant decrease in water absorption, that is, (58%–55%) at the level of 20% wheat flour replacement with chickpea flour. These researchers further observed a nonsignificant change in water absorption capacity of composite flour at 10% supplementation thereby suggesting an optimum level of supplementation to design a quality product.

**TABLE 2:** Farinographic and mixographic measurements of spinach powder supplemented Chapattis

Parameters	Spinach powder supplemented chapatti premixes						
	*T*_0_	*T*_1_	*T*_2_	*T*_3_	*T*_4_	*T*_5_	*T*_6_
Farinographic measurements							
Water absorption (%)	62.67 ± 0.78a	58.64 ± 0.50bc	59.79 ± 0.32b	57.98 ± 0.04c	57.49 ± 0.71cd	56.22 ± 0.95de	54.89 ± 0.79e
Dough stability (min)	13.21 ± 0.00a	12.49 ± 0.06b	12.02 ± 0.01bc	11.91 ± 0.18c	11.60 ± 0.37c	11.10 ± 0.34d	10.67 ± 0.01d
Dough development (min)	5.56 ± 0.16a	4.12 ± 0.16d	4.67 ± 0.00c	4.71 ± 0.06bc	4.93 ± 0.08b	5.44 ± 0.01a	5.60 ± 0.06a
Mixographic measurements							
Peak height (BU)	48.59 ± 0.51g	49.16 ± 0.06f	50.43 ± 0.00e	51.43 ± 0.16d	55.39 ± 0.08c	62.22 ± 0.14b	66.39 ± 0.08a
Peak time (min)	5.37 ± 0.04a	5.02 ± 0.01b	4.53 ± 0.02c	4.35 ± 0.01d	4.03 ± 0.01e	3.97 ± 0.01f	3.44 ± 0.01g

Note

Values are means ± S.D. Values having identical lettering in each column are nonsignificant at *p* < .05. *T*_0_ = 100% whole wheat flour (control), *T*_1_ = 2.5% spinach powder, (SP), *T*_2_ = 5% SP, *T*_3_ = 7.5% SP, *T*_4_ = 10% SP, *T*_5_ = 15% SP, *T*_6_ = 20% SP.

### 3.3 Nutritional composition of dehydrated spinach and value‐added product

Spinach powder dried to moisture contents at ~8% was shown to carry a considerable amount of fiber (8.2%), ash (2.9%), and protein (19.2%). A relatively lower amount of fiber, ash, and protein contents, that is, 2.4%, 0.2%, and 8.6%, respectively, were recorded for wheat flour (Table 3). Increased variability was recorded for protein (21%–32%) and inorganic contents (10%–18%) of spinach powder in comparison with retrospective studies (Galla et al., 2017; Khan et al., 2015). Similarly, variation in ash (%) and protein (%) contents may be linked to the varietal differences, geographical conditions, and degree of dehydration or total solid contents in dehydrated plant material. Enriching wheat flour with nutrient‐dense ingredients like vegetable powder may result in improved nutritional status of the finished products. Significant (*p* < .05) effect of spinach powder supplementation was witnessed on the nutritional composition of unleavened flatbread, that is, *chapattis* (Table 3). The results suggested significant improvement in protein, ash, and fiber contents of composite *chapattis* with values ranging from 8.0% to 9.3%, 0.3 to 0.9%, and 2.4 to 4.1%, respectively. A significant increase (*p *< .05) in protein, ash, and fiber contents of wheat–spinach composite *chapattis* might be ascribed to spinach powder supplementation which had itself delivered higher amounts of these constituents (Table 3). A notable decrease in moisture contents of spinach powder supplemented *chapattis* was also noted which hinted at the lower water holding capacity of the spinach powder to be the reason as compared to wheat flour. Results of the present research are in agreement with the previous findings of Seleem and Omran (2014) who reported partial replacement of wheat flour with sorghum flour and noncereals crops like beans to anticipate comparatively higher levels of fiber, ash, and protein in supplemented *chapattis,* that is, 9.2%–10.2%, 1.6%–2.1%, and 0.4%–0.8%, respectively, while relatively lower values of the aforesaid nutrients were recorded for sorghum supplemented chapattis, that is, 9.0%–9.3%, 1.6%–1.7%, and 0.4%–0.5%.

**TABLE 3:** Proximate composition of spinach powder supplemented Chapattis (g/100g)

Parameters	Samples						
	*T*_0_	*T*_1_	*T*_2_	*T*_3_	*T*_4_	*T*_5_	*T*_6_
Moisture	37.65 ± 0.04a	37.25 ± 0.36a	35.25 ± 1.77ab	34.00 ± 0.00ab	32.98 ± 1.06bc	31.60 ± 0.56cd	29.25 ± 1.77d
Ash	0.31 ± 0.01g	0.39 ± 0.00f	0.47 ± 0.01e	0.55 ± 0.03d	0.63 ± 0.03c	0.77 ± 0.02b	0.92 ± 0.02a
Protein	8.03 ± 0.02g	8.85 ± 0.01f	9.33 ± 0.04e	9.78 ± 0.04d	10.26 ± 0.03c	11.25 ± 0.03b	12.21 ± 0.05a
Fat	1.66 ± 0.04a	1.60 ± 0.00a	1.52 ± 0.03b	1.32 ± 0.03c	1.24 ± 0.03d	1.18 ± 0.01d	1.07 ± 0.02e
Fiber	2.44 ± 0.01g	2.64 ± 0.01f	2.86 ± 0.02e	3.05 ± 0.01d	3.27 ± 0.02c	3.78 ± 0.17b	4.09 ± 0.04a
Carbohydrates	49.91 ± 0.04c	49.27 ± 0.34c	50.57 ± 1.74bc	51.20 ± 0.04b	51.63 ± 0.95ab	51.41 ± 0.32ab	52.46 ± 1.68a
Caloric value (Kcal/100g)	247.53 ± 0.29e	247.80 ± 1.36e	254.23 ± 6.79d	257.18 ± 0.25bc	259.73 ± 4.18c	262.40 ± 1.53b	269.53 ± 6.77a

Note

Values are means ± S.D. Values having identical lettering in each column are nonsignificant at *p* < .05. *T*_0_ = 100% whole wheat flour (control), *T*_1_ = 2.5% spinach powder, (SP), *T*_2_ = 5% SP, *T*_3_ = 7.5% SP, *T*_4_ = 10% SP, *T*_5_ = 15% SP, *T*_6_ = 20% SP.

### 3.4 Mineral contents of dehydrated spinach and value‐added product

Comparing with wheat flour carrying Ca (41 mg/100 g) and Fe (3.8 mg/100 g), spinach powder exhibited significantly higher amounts (*p *< .05) of Ca and Fe, that is, 1304 mg/100g and 40mg/100g, respectively. Wheat flour is not considered a good source of dietary iron and calcium which is exacerbated by whole grain processing and refining leading to a significant loss of the minerals’ reserves. Contrarily, vegetables hold a tremendous amount of micronutrients when offered in raw or as dehydrated powders. Results of the current study validate earlier reports wherein dehydrated spinach was reported to contain Ca 1020‐1336 mg/100g and Fe 30‐69 mg/100g (Galla et al., 2017; Khan et al., 2015).

A significant increase (*p *< .05) in levels of micro‐ and macrominerals in composite flour *chapattis* was observed indicating spinach powder supplementation to be directly associated with improved minerals profile of the final product (Table 4). Wheat flour enrichment with spinach powder (20%) increased K, Ca, and Fe contents of *chapattis* from 448 to 494 mg/100g, 40 to 301 mg/100g, and 3.3 to 11.6 mg/100g, respectively, and such an elevation in minerals concentration of *chapattis* is attributed to a better micronutrients profile of spinach powder (Table 4). Our findings for the mineral composition of the composite *chapattis* are in line with those of Seleem and Omran (2014) who demonstrated a significant increase in Ca, Fe, and Zn contents of bean flour supplemented *chapattis,* that is, 15.3 to 21.6 mg/100g, 6.0 to 7.1 mg/100g, and 0.9 to 1.2 mg/100g, respectively.

**TABLE 4:** Mineral composition of spinach powder supplemented Chapattis (mg/100g)

Parameters	Spinach powder supplementation levels								
	Whole wheat flour	Spinach powder	*T*_0_	*T*_1_	*T*_2_	*T*_3_	*T*_4_	*T*_5_	*T*_6_
Sodium	5.06 ± 0.08b	98.20 ± 0.78a	5.34 ± 0.32g	8.10 ± 0.14f	10.47 ± 0.04e	12.90 ± 0.03d	15.27 ± 0.08c	20.16 ± 0.06b	25.37 ± 0.40a
Calcium	40.80 ± 0.35b	1,303.90 ± 3.78a	39.83 ± 0.05g	72.71 ± 0.25f	105.54 ± 0.48e	137.81 ± 0.08d	170.70 ± 0.24c	235.65 ± 0.29b	301.26 ± 0.11a
Potassium	445.50 ± 0.71a	233.38 ± 1.34b	448.25 ± 1.77g	452.01 ± 1.14f	458.13 ± 0.69e	464.82 ± 0.55d	469.84 ± 0.58c	481.99 ± 0.18b	493.86 ± 0.53a
Iron	3.78 ± 0.02b	40.36 ± 0.21a	3.27 ± 0.02f	4.16 ± 0.20e	5.60 ± 0.42d	6.51 ± 0.28c	7.16 ± 0.22c	9.59 ± 0.39b	11.62 ± 0.41a
Zinc	3.91 ± 0.02b	13.38 ± 0.23a	3.44 ± 0.14g	3.91 ± 0.04f	4.28 ± 0.09e	4.58 ± 0.04d	4.89 ± 0.00c	5.64 ± 0.09b	6.35 ± 0.14a

Note

Values are means ± S.D. Values having identical lettering in each column are nonsignificant at *p* < .05. *T*_0_ = 100% whole wheat flour (control), *T*_1_ = 2.5% spinach powder, (SP), *T*_2_ = 5% SP, *T*_3_ = 7.5% SP, *T*_4_ = 10% SP, *T*_5_ = 15% SP, *T*_6_ = 20% SP.

### 3.5 Effect of spinach powder supplementation on textural properties of *chapattis*

Color measurements of dried spinach substituted *chapattis* are presented in Table 5. Lightness (L*) and redness (*) values of composite *chapattis* significantly decreased (*p < *.05) in a dose‐dependent manner from 70 to 52 and 2.9 to 1.0, respectively, with an increasing level of spinach powder supplementation. Reduction in L* values depicts that *chapattis* are darker in color. In the same way, yellowness (b*) values of chapattis increased from 18.8 to 27.8 with an increasing level of spinach powder supplementation. Color variations in wheat‐based *chapattis* are generally attributed to Maillard reaction during baking, however; higher incidence of variability in color profile of spinach powder supplemented chapattis may better be correlated with spinach pigments like carotenoids. Substituting wheat flour with spinach powder significantly influenced the color index of the baked product that may lead to a significant decline in securing consumer acceptability score. Replacing wheat flour with pigmented grains (sorghum) and dark‐colored vegetables like spinach at levels beyond 10% and 30%, respectively, have been documented to attribute the decline in consumer acceptability score of baked goods. Comparable findings were reported by several researchers, wherein L* values were decreased on the addition of spinach powder in extrusion products (Galla et al., 2017; Singh et al., 2015). Similarly, other studies reported similar findings for b* values in *chapattis* made with jering seeds flour (Cheng & Bhat, 2015).

**TABLE 5:** Effect of spinach powder supplementation on color values, instrumental texture, and puffed height of Chapattis

Parameters	Spinach powder supplementation levels						
	*T*_0_	*T*_1_	*T*_2_	*T*_3_	*T*_4_	*T*_5_	*T*_6_
Hunter lab color							
Lightness (L*)	69.83 ± 0.69a	67.35 ± 0.53b	64.43 ± 0.00c	62.98 ± 0.94c	59.04 ± 0.71d	57.04 ± 0.71e	51.66 ± 0.77f
Redness (a*)	2.93 ± 0.08ab	2.77 ± 0.14b	2.77 ± 0.01b	2.95 ± 0.05a	2.22 ± 0.01c	1.60 ± 0.07d	0.99 ± 0.02e
Yellowness (b*)	18.87 ± 0.16f	19.22 ± 0.14f	20.07 ± 0.07e	22.54 ± 0.16d	23.65 ± 0.16c	25.38 ± 0.54b	27.82 ± 0.23a
Chroma	19.09 ± 0.16f	19.42 ± 0.16f	20.26 ± 0.07e	22.74 ± 0.15d	23.75 ± 0.16c	25.43 ± 0.54b	27.47 ± 0.28a
Hue angle (θ°)	81.19 ± 0.16f	81.80 ± 0.35e	82.14 ± 0.01de	82.56 ± 0.17d	84.65 ± 0.06c	86.38 ± 0.23b	87.95 ± 0.06a
Instrumental texture analysis							
Hardness (g)	22.39 ± 0.00g	43.49 ± 0.00f	43.93 ± 0.00e	48.80 ± 0.02d	82.43 ± 0.04c	86.13 ± 0.00b	89.55 ± 0.48a
Springiness (g)	66.70 ± 0.20f	60.86 ± 0.01g	95.76 ± 0.13e	102.50 ± 0.25d	118.61 ± 0.09c	137.85 ± 0.12b	147.48 ± 0.09a
Chewiness (g)	67.02 ± 0.03g	79.22 ± 0.30f	95.16 ± 0.23e	116.28 ± 0.40d	126.38 ± 0.54c	132.05 ± 0.07b	138.23 ± 0.32a
Puffed Height (cm)	5.45 ± 0.01a	5.02 ± 0.04b	4.97 ± 0.09c	4.92 ± 0.05d	4.55 ± 0.06e	4.35 ± 0.12f	4.03 ± 0.20g

Note

Values are means ± S.D. Values having identical lettering in each column are nonsignificant at *p* < .05. *T*_0_ = 100% whole wheat flour (control), *T*_1_ = 2.5% spinach powder, (SP), *T*_2_ = 5% SP, *T*_3_ = 7.5% SP, *T*_4_ = 10% SP, *T*_5_ = 15% SP, *T*_6_ = 20% SP.

Hardness and chewiness of spinach powder supplemented *chapattis* significantly increased (*p *< .05) from 22 to 90 g and 67 to 138 g, respectively, with increasing spinach powder supplementation level (Table 5). A study by Singh et al. (2015) reported higher fiber and protein contents of spirulina flour to mark a significant increase in hardness of wheat‐based spirulina flour supplemented biscuits. Results on textural properties observed in the present study are in agreement with the findings of an earlier report, wherein an increase in hardness was recorded for biscuits supplemented with spinach powder at 15% supplementation level (Galla et al., 2017). Likewise, substituting wheat flour with jering seed at 0 to 100% substitution level significantly increased hardness in *chapattis,* that is, 332.4 g to 1627.5 g (Cheng & Bhat, 2015).

Puffing of *chapattis* is a desirable quality attribute that significantly influences consumers’ preference (Wani et al., 2016). Textural profiling of spinach powder supplemented *chapattis* revealed a significant decline in puffing quality of the baked good and puffing height score declined from 5.45 to 4.03 cm at 0 to 20% supplementation (Table 5). Reduction in *chapattis* puffing height might be attributed to higher amounts of the dietary fibers and nongluten proteins of spinach powder.

### 3.6 Effect of spinach powder supplementation on the sensory acceptability of chapattis

Data extracted from the organoleptic acceptability study of spinach powder supplemented composite chapattis are presented in Table 6. Acceptable scores for various sensory variables of spinach powder supplemented *chapattis* were recorded by the sensory panelists at 7.5% supplementation. The results delineated a significant decline in sensory scoring with increasing levels of spinach powder supplementation, however; 10 to 20% substitution of wheat flour with spinach powder resulted in undesirable sensory attributes when compared with the normal control. Spinach‐based products, that is, biscuits, have already been reported for sensory acceptability wherein researchers documented better consumer acceptability scores at 5% spinach powder supplementation (Galla et al., 2017). Another study by Khan et al. (2015) reported an organoleptically acceptable sensory score for *chapattis* developed at 5%spinach powder supplementation.

**TABLE 6:** Effect of spinach powder supplementation on sensory acceptability of Chapattis

Parameters	Spinach powder supplementation levels						
	*T*_0_	*T*_1_	*T*_2_	*T*_3_	*T*_4_	*T*_5_	*T*_6_
Appearance	7.76 ± 0.16a	6.71 ± 0.13bc	6.36 ± 0.30c	6.91 ± 0.34b	4.72 ± 0.40d	3.86 ± 0.47e	3.84 ± 0.72e
Color	7.81 ± 0.28a	6.58 ± 0.34b	6.63 ± 0.29b	6.86 ± 0.35b	4.86 ± 0.47c	4.29 ± 0.72d	4.07 ± 0.47d
Folding ability	7.69 ± 0.23a	6.51 ± 0.21c	6.41 ± 0.35c	7.11 ± 0.48b	4.85 ± 0.39d	4.15 ± 0.49e	3.99 ± 0.60e
Taste	7.54 ± 0.21a	6.65 ± 0.23b	6.52 ± 0.31b	6.84 ± 0.35b	4.71 ± 0.41c	3.84 ± 0.53d	3.77 ± 0.86d
Texture	7.73 ± 0.21a	6.52 ± 0.21c	6.63 ± 0.33c	7.18 ± 0.37b	4.95 ± 0.36d	4.12 ± 0.44e	4.02 ± 0.69e
Overall effect	7.82 ± 0.25a	6.77 ± 0.11bc	6.47 ± 0.30c	6.92 ± 0.22b	4.94 ± 0.32d	4.02 ± 0.43e	3.67 ± 0.61e

Note

Values are means ± S.D. (*n* = 30). Values having identical lettering in each column are nonsignificant at *p* < .05. *T*_0_ = 100% whole wheat flour (control), *T*_1_ = 2.5% spinach powder, (SP), *T*_2_ = 5% SP, *T*_3_ = 7.5% SP, *T*_4_ = 10% SP, *T*_5_ = 15% SP, *T*_6_ = 20% SP.

## 4 CONCLUSIONS

Growing micronutrient deficiencies especially in lower‐middle‐income countries are a leading cause of increased disease burden. Novel intervention strategies to confront the challenges associated with malnutrition and food insecurities are imperative. Archetypical consumption of vegetables is now transitioning to novel approaches to foster their enhanced intake. The application of dehydrated vegetables is being widely practiced in a variety of recipes and food preparations. Spinach powder is considered a valuable source of dietary fibers, micronutrients, and bioactive compounds that can ameliorate several nutritional and health disorders. The present investigation confirmed the addition of spinach powder in wheat flour‐based baked goods at ~ 10% level of supplementation not to contribute undesirable sensory attributes to unleavened bread, that is, *chapattis*. Our findings conclude that spinach powder supplementation at 7.5% could be a viable and practical approach to develop value‐added baked goods. The results further indicate that the utilization of green leafy vegetables as powder formulae or premixes could help abridge nutritional inadequacies and food insecurities cost‐effectively among vulnerable population groups.

## CONFLICT OF INTEREST

Authors declare to not have any conflict of interest.

## ETHICAL APPROVAL

This does not involve human or animal modeling.
